# Long-term outcome of definitive radiotherapy for cervical esophageal squamous cell carcinoma

**DOI:** 10.1186/s13014-018-0957-6

**Published:** 2018-01-18

**Authors:** Katsuyuki Sakanaka, Yuichi Ishida, Kota Fujii, Satoshi Itasaka, Shin’ichi Miyamoto, Takahiro Horimatsu, Manabu Muto, Takashi Mizowaki

**Affiliations:** 1 Department of Radiation Oncology and Image-Applied Therapy, Graduate School of Medicine, Kyoto University, Kyoto, Japan; 2 Department of Gastroenterology and Hepatology, Graduate School of Medicine, Kyoto University, Kyoto, Japan; 3 Department of Therapeutic Oncology, Graduate School of Medicine, Kyoto University, Kyoto, Japan

**Keywords:** Esophageal Neoplasms, Radiotherapy, Conformal, Long-term outcome, Hypothyroidism, Cardiovascular disease

## Abstract

**Background:**

The aim of this study was to identify the long-term clinical outcome of definitive radiotherapy using three-dimensional conformal radiotherapy (3DCRT) for cervical esophageal squamous cell carcinoma (CESCC).

**Methods:**

We retrospectively reviewed the medical records of 30 patients with CESCC [clinical stage I/II/III/IV(M1LYM); 3/2/12/13] (TNM 7th edition) who underwent definitive radiotherapy using 3DCRT between 2000 and 2014 in our institution. The median prescribed dose for the gross tumor and metastatic lymph nodes was 60 Gy. Twenty-six patients underwent elective nodal irradiation for the neck node levels III, IV, and VI and for upper mediastinal lymph nodes with a median dose of 40 Gy. Twenty-six patients underwent concurrent chemotherapy. Initial disease progression sites, locoregional control (LRC) rate, overall survival (OS) rate, and toxicities were retrospectively evaluated. A univariate analysis was performed to identify prognostic factors.

**Results:**

With a median follow-up of 110 months, the 5- and 10-year LRC rates were 43.7% and 37.4%, respectively. The 5- and 10-year OS rates were 48.3% and 40.2%, respectively. Locoregional, distant and both area accounted for 83%, 6% and 11% of the initial progression sites. Unresectable status and M1LYM were significantly associated with poor LRC (*p* < 0.05) and OS (*p* < 0.05). Grade 3 acute non-hematological toxicity occurred in 13.3% of patients. During the follow-up, patients without any disease progression did not need a permanent gastrostomy tube or tracheostomy. Late toxicity events, including hypothyroidism and cardiovascular disease, were observed; 5- and 10-year cumulative incidence rates of grade 2 hypothyroidism and ≥grade 3 cardiovascular disease were 31.6% and 62.5%, and 17.5% and 21.3%, respectively.

**Conclusions:**

Definitive radiotherapy yields a cure for patients with CESCC while preserving their laryngopharyngeal function. The poor LRC rate in the advanced stage needs to be overcome for a better prognosis. As the incidence of radiation-induced hypothyroidism and cardiovascular disease was not low, long-term survivors should be followed up for these symptoms.

## Background

Radiotherapy is a definitive treatment for cervical esophageal squamous cell carcinoma (CESCC). The total doses of definitive radiotherapy for CESCC range from 50 to 70 Gy [1–10]. Chemotherapy was concurrently administered in 28%–100% of patients [1–5, 7–12]. The major progression site had some discrepancy between reports, including the locoregional area in eight reports [2–6, 9–11] and a distant area in three reports [1, 7, 12].

The 2-year local failure free survival, the 5-year locoregional control (LRC) and the 5-year overall survival (OS) rates of patients undergoing definitive radiotherapy for CESCC were 69.9%–74.5% [12, 13], 47% [3] and 25%–55% [1–3, 6], respectively. Advanced stage tumors [2, 12], hoarseness [7], and weight loss [12] are associated with a poor prognosis. Grade 3–4 acute non-hematological toxicity has been reported to be 8.3%–24.5% [2, 4, 5, 7, 10]. Symptomatic late toxicity related to radiotherapy has been described in five reports, including esophageal stricture [1, 4], Lhermitte’s sign [1], hypothyroidism [2], and brachial plexus injury [8], whereas two reports described no symptomatic late toxicity events [3, 11].

Surgery is the established treatment modality for CESCC. The 5-year OS rate was 33%–47% [14–16]. However, surgical procedures for CESCC often require laryngopharyngectomy. Avoiding laryngectomy needs no tumor invasion to larynx, trachea and inlet of esophagus. Those situations are rare in patients with CESCC. In three medical reports on patients with CESCC, 33%–92% patients underwent laryngopharyngectomy [14, 16, 17]. Marmuse et al. performed larynx preserving surgery for 67% of patients with CESCC but the percentage of incomplete resection was not low; the macroscopic residual tumors were left among 37% of patient who underwent larynx preserving surgery [16]. Preoperative treatment may have improved the preservation of the larynx; however, minor and major complications occurred in 74.3% of patients and an additional operation was necessary for 29.4% of patients [15].

The reports of definitive radiotherapy for CESCC are increasing. Two reports have suggested that definitive radiotherapy yields OS rates that are comparable with those after surgery [5, 13]. However, previous reports on radiotherapy for CESCC have included limited number of patients [1–12]. In addition, long-term follow-up reports are sparse. Importantly, the median follow-up in previous reports did not reach 5 years [1–12], with the exception of one study [3]. Long-term follow-up outcomes are necessary for establishing the role of definitive radiotherapy for CESCC. It will help in decreasing concerns of late adverse events related to radiotherapy, which can cause non-cancer-related death in patients with thoracic esophageal cancer [18, 19].

The aim of the current study was to identify the long-term clinical outcome of definitive radiotherapy for CESCC with a median follow-up of 110 months in a single center experience.

## Methods

### Inclusion criteria

This study (R1048) was approved by the institutional review board of the Kyoto university on March 16, 2017, and was conducted in accordance with the Declaration of Helsinki and Japanese ethical guidelines for epidemiological research. Inclusion criteria were as follows:

1. the primary esophageal squamous cell carcinoma existed in the cervical esophagus
2. ≥50 Gy was prescribed for all gross tumors and metastatic lymph nodes with a definitive intent
3. radiotherapy was initiated from December, 2000 to December, 2014

Thirty patients met the inclusion criteria of this study.

### Collected clinical information

This study collected the following information from medical records: age; gender; Eastern Cooperative Oncology Group performance status (PS); smoking habits; comorbidities; clinical stage based on the seventh edition of the TNM classification for esophageal cancer of the Union for International Cancer Control criteria; tumor location; image findings including computed tomography (CT) of the chest, abdomen, and pelvis; endoscopic findings; resectability; details of radiotherapy and chemotherapy; and side effects. The tumor location was categorized into cervical esophagus alone, cervical esophagus with a hypopharyngeal invasion, cervical esophagus with an upper thoracic esophageal invasion, and cervical esophagus with invasion in both areas. The patient characteristics are listed in Table 1. A multidisciplinary oncology team (surgeon, gastroenterologist, medical oncologists, and radiation oncologist) diagnosed the clinical T-, N- and M-status on the basis of a comprehensive assessment of the patient’s physical and imaging examinations. Clinical T4 staging was mainly based on CT findings. Only one patient was diagnosed as cT4 based on the bronchoscope finding. The invasion of the primary tumor or metastatic lymph nodes to the trachea or the major vascular encasement was considered unresectable. The locations of the non-regional lymph nodal metastasis (M1LYM) were the supraclavicular lymph nodes in eleven patients, cervical level III lymph nodes in one patient, and both area in one patient, respectively. They were all included by ENI and boost radiotherapy. A gastrostomy tube was placed into three patients before radiotherapy and into two patients during radiotherapy. A tracheostomy tube was placed before radiotherapy into one patient with airway stenosis because of a primary esophageal tumor.

**Table 1:** Patient characteristics

Parameters	
Median age	65 (interquartile range 60–69)
Female/Male	5/25
Performance status (0–1/2)	26/4
Current smoker/others	19/11
Current drinker/others	26/4
Medications for cardiovascular disease/none	17/13
Clinical stage (I/II/III/IV) ^a^	3/2/12/13
T status (1/2/3/4)	3/4/6/17
N status (0/1/2/3)	9/16/5/0
M status (0/1LYM) ^b^	17/13
Resectable/unresectable^c^	10/20
Concurrent chemotherapy (with/without)	26/4

^a^Classification of the clinical stage was based on the seventh edition of the TNM classification for esophageal cancer

^b^M1LYM means supraclavicular or cervical lymph nodal metastasis, which was included in the irradiation fields

^c^The invasion of the primary tumor or metastatic lymph nodal metastasis to the trachea or artery

### Localization and target delineation of the primary tumor and metastatic lymph nodes

All radiotherapy plans were created using CT simulation with patients in a supine position. We contoured the primary tumor and metastatic lymph nodes as gross target volumes (GTVs) based on findings of endoscopy and CT [20]. We created the clinical target volume of the primary tumor (CTVp) by adding a 2 cm margin in the craniocaudal direction and a 0.5 cm margin in the radial direction of the primary tumor. The clinical target volume of the metastatic lymph nodes (CTVlym) was created by adding a 0.5 cm margin. The volume of CTVp and CTVlym with a 0.5 cm margin was defined as the planning target volume (PTV).

### Radiotherapy planning and dose delivery

A dose was delivered 5 days a week by a linear accelerator using 6–15-MV photon beams for all patients. The median overall treatment time for radiotherapy was 44 days [Interquartile range (IQR) 42–46].

Among 30 patients, 26 underwent elective nodal irradiation (ENI). ENI included the cervical lymph nodal region: levels III, IV, and VI [21] and paraesophageal and paratracheal lymph nodal regions of the upper mediastinum. The caudal extent of ENI was approximately 4 cm below the carina. Anterior–posterior and posterior–anterior irradiation fields were used for ENI. The median dose of ENI was 40 Gy (IQR 40–40). Then boost radiotherapy was initiated using anterior oblique wedged pair two fields, opposed parallel two fields, or unparalleled four fields. Its fields included PTV with 0.5 cm leaf margins except in the dorsal direction to spare the spinal cord. The dorsal leaf margin in the boost field was set to completely include GTVs. The median minimal dorsal leaf margin for GTVs in the boost field was 0.5 cm (IQR 0–1.0). The median total dose of ENI with boost radiotherapy was 60 Gy (IQR 60.0–60.0).

The other four patients underwent definitive radiotherapy without ENI. The median dose was 60 Gy (IQR 60.0–61.5). Anterior oblique wedged pair two fields or unopposed three fields were used to include PTV with 0.5 cm leaf margins except in the dorsal direction. The dorsal leaf margin of each field was reduced, and the median minimal dorsal leaf margin for GTVs was 0.5 cm (IQR 0–1.0).

### Details of chemotherapy

Twenty-six patients underwent concurrent chemotherapy. It was based on the platinum analogues (cisplatin or nedaplatin) and the continuous 5-fluorouracil drip. Twenty-one patients received two cycles of cisplatin (70–75 mg/m^2^ per day) on day 1 and 5-fluorouracil (700–1000 mg/m^2^ per day) on day 1 for 4 or 5 days at the interval of 4 weeks [22–24]. One patient received nedaplatin (90 mg/m^2^ per day) on day 1, 29 and 5-fluoruracil (800 mg/m^2^ per day) on days 1–4, 29–32. Two patients received low dose cisplatin and 5-fluorouracil administration; one patient received two cycles of cisplatin (7 mg/m^2^ per day for 10 days) and 5-fluorouracil (250 mg/m^2^ per day for 14 days) at the interval of 4 weeks, and the other patient received cisplatin (4 mg /m^2^ per day) and 5-fluorouracil (200 mg/m^2^ per day) on five days of each week during radiotherapy. Two patients received single agent chemotherapy; one patient received two cycles of 5-fluorouracil drip (700 mg/m^2^/day) for 4 days at the interval of 4 weeks, and the other patient received cisplatin (80 mg/m^2^ per day) on days 1 and 29.

### Follow-up

The response of the tumor was initially evaluated at 4–6 weeks after the final session of radiotherapy. Progression of the primary tumor was evaluated by endoscopy. Progression of regional lymph nodes and distant metastasis was evaluated by CT. 18F–fluorodeoxyglucose positron emission tomography was used as a supportive modality. These treatments, together with physical examinations, were repeated every 1–3 months until a complete response or disease progression was confirmed. Following confirmation of a complete response, the follow-up interval was changed to 3–6 months.

### Patten of recurrence, LRC, OS, and side effects

Here we categorized initial progression sites into locoregional areas, distant areas, or both. LRC and OS were estimated using the Kaplan–Meier test. LRC events were locoregional progression and censored on the date that LRC was most recently verified. Any esophageal tumors which progressed within irradiation fields were counted as locoregional progression events. Metachronous superficial esophageal tumors out of radiation fields were not counted as locoregional progression events. OS events included death from any cause and censored at the final follow-up.

Side effects within 90 days after the initial day of chemoradiotherapy were judged as an acute toxicity. Side effects occurring 91 days after the initiation of chemoradiotherapy were judged as late toxicity. They were graded using the Common Terminology Criteria for Adverse Events, version 4.0.

### Statistical analysis

Differences between LRC and OS among the categorical variables were analyzed by the log-rank test. Categorical variables included gender (female vs. male), age (≤65 vs. > 65 years), PS (0–1 vs. 2), T status (T1–3 vs. T4), N status (N0 vs. N1–3), M status (M0 vs. M1LYM), and resectability (resectable vs. unresectable). All statistical tests were two-sided. Categorical variables with *p* values of < 0.05 were statistically significant. Log-rank tests were conducted using GraphPad software (ver. 5.03; GraphPad Software, San Diego, CA, USA).

Cumulative incidence rates of late toxicity events were calculated using the cumulative incidence model. Death events were treated as a competing risk for calculating the cumulative incidence of late toxicity events. To calculate the cumulative incidence of radiation-induced hypothyroidism, death events and salvage surgery including thyroidectomy were treated as a competing risk. Statistical analyses were performed using EZR (Saitama Medical Center, Jichi Medical University, Saitama, Japan), a graphical user interface for R (The R Foundation for Statistical Computing, Vienna, Austria). More precisely, it is a modified version of the R commander that includes statistical functions frequently used in biostatistics [25].

## Results

### LRC, OS, and pattern of recurrence

**Fig. 1:**
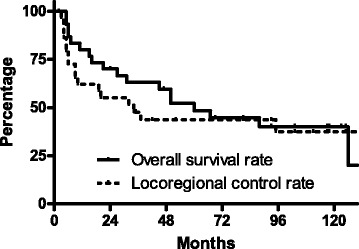
Kaplan–Meier curves for locoregional control (LRC) and overall survival (OS) rates in the patients

The median follow-up for censored patients was 110 months (IQR 78–124). Five- and 10-year LRC rates were 43.7% [95% confidence interval (CI), 25.2–60.8] and 37.4% (95% CI, 18.9–56.0), respectively. Five- and 10-year OS rates were 48.3% (95% CI, 29.4–65.0) and 40.2% (95% CI, 22.1–57.6), respectively (Fig. 1).

Eighteen patients experienced disease progression. The median time to disease progression was 9 months (IQR 5–20) after the initial day of radiotherapy. Seventeen patients experienced locoregional progression initially, including only tumor bed (primary tumor and metastatic lymph nodes) in 14 patients, tumor bed plus distant metastasis in 2 patients, and only regional lymph nodal progression in 1 patient. Distant metastasis (lung) without locoregional progression occurred in one patient. Four patients underwent salvage surgery: total pharyngolaryngoesophagectomy for 2 patients and lymph nodal dissection for 2 patients. One metachronous esophageal superficial tumor was detected in the follow-up, which was successfully resected by endoscopic resection 62 months after the initial day of radiotherapy.

### Prognostic factors for OS and LRC

**Fig. 2:**
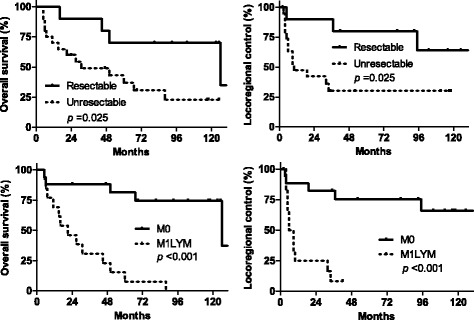
Kaplan–Meier curves for LRC and OS rates in patients with resectable and unresectable statuses and in those with M0 and M1LYM. Abbreviations: M0 means the absence of non-regional lymph nodal metastasis; M1LYM means the existence of non-regional lymph nodal metastasis

Unresectable and M1LYM statuses were associated with a poor OS rate [*p* = 0.025; hazard ratio (HR) = 3.05 (95% CI, 1.15–8.08) and *p* < 0.0001; HR = 11.5 (95% CI, 3.87–34.0), respectively]. The 5-year OS rate was 70% (95% CI, 32.9–89.2) for patients with resectable status vs. 36.8% (95% CI, 16.0–58.0) for those with unresectable status and 81.4% (95% CI, 52.6–93.6) for those with M0 vs. 7.7% (95% CI, 0.5–29.2) for those with M1LYM (Fig. 2). Statistically significant differences were not observed in age (*p* = 0.56), gender (*p* = 0.72), PS (*p* = 0.51), T status (*p* = 0.11), and N status (*p* = 0.59) for OS.

Unresectable and M1LYM statuses were also associated with a poor LRC [*p* = 0.025, HR = 3.23 (95% CI, 1.16–8.97) and *p* = 0.0001, HR = 9.66 (95% CI, 3.02–30.9), respectively]. The 5-year LRC rate was 80% (95% CI, 40.9–94.6) for patients with resectable status vs. 30.1% (95% CI, 11.5–51.3) for those with unresectable status and 75.5% (95% CI, 46.9–90.1) for patients with M0 vs. 8.3% (95% CI, 0.5–31.1) for those with M1LYM (Fig. 2). A statistically significant difference was not observed in age (*p* = 0.74), gender (*p* = 0.29), PS (*p* = 0.88), T status (*p* = 0.41), or N status (*p* = 0.30) for LRC.

### Acute and late toxicity

Grade 4 acute hematological (neutropenia) occurred in one patient and ≥grade 3 acute non-hematological toxicity event related to radiotherapy (esophagitis, anorexia, and dysphagia) occurred in four patients.

Among the 30 patients, six experienced ≥grade 3 cardiovascular disease (atrial fibrillation in one patient, arteriosclerosis obliterans in one patient, unstable angina in three patients, and acute coronary disease in one patient). One patient with atrial fibrillation died of stroke without recurrence of CESCC. The median onset time of cardiovascular disease after radiotherapy was 33.5 months (IQR 19–47).

The 5- and 10-year cumulative incidence rates of ≥grade 3 cardiovascular events were 17.5% (95% CI, 6.1–33.7) and 21.3% (95% CI, 8.3–38.2), respectively. Symptomatic pericarditis and pericardial effusion definitely, possibly, and probably related to radiotherapy or symptomatic radiation pneumonitis were not observed during the follow-up for all included patients. Among 13 patients without any disease progression, ≥grade 3 esophageal stricture or ≥grade 1 fistula formations as late toxicity events were not observed. None of them needed permanent gastrostomy feeding tubes or temporary tracheostomy tubes because of radiation-related late toxicity.

The thyroidal function of 17 patients was examined by venous blood sampling after radiotherapy. Grade 2 hypothyroidism occurred in 14 patients during the follow-up. Among them, hypothyroidism in three patients resulted from total thyroidectomy during salvage surgery for the locoregional recurrence of CESCC. The median time to detect grade 2 hypothyroidism was 57 months (IQR 33–96) after the initial day of radiotherapy, with the exception of three patients undergoing thyroidectomy. The 5- and 10-year cumulative incidence rates of hypothyroidism were 31.6% (95% CI, 15.4–49.2) and 62.5% (95% CI, 29.6–83.3), respectively.

## Discussion

Definitive radiotherapy using three-dimensional conformal radiotherapy (3DCRT) yielded a cure for 40% patients with CESCC, preserving their laryngopharyngeal function. LRC and OS rates of radiotherapy from a previous report [3] and this report are comparable to those after surgery [14–16]. The local-regional area (tumor bed) was the major progression area. The unresectable status and existence of non-regional lymph nodal metastasis were detrimental to LRC and OS. Late adverse events were almost negligible except for radiation-induced hypothyroidism and cardiovascular disease.

Three reports on radiotherapy for CESCC concluded that distant metastasis was the major progression site. Among them, two reports counted all failures in one patient regardless of the history of previous failures [7, 12]. Therefore, the proportion of distant metastasis increased in these studies. Another report showed complete response [91% (31 of 34 patients)] after radiotherapy, where six patients developed distant metastases [1]. Most of the included patients (27/34) in this study had an early resectable status (T1-2N0M0). Therefore, good LRC rates could have been achievable as observed in the current study. However, in that study, routine endoscopy and radiological examination during follow-up were not performed. There were concerns that it possibly missed local-regional progression. The 5-year LRC rate was less than 50% except for that indicated by one report [1]. Although further studies are necessary to conclude the major progression area, local-regional control needs to be improved for a cure. The control of gross tumors for CESCC should focus on the use of radiotherapy in the future.

In the current study, we minimized the dorsal leaf margins of boost fields for GTVs to spare the spinal cord. It was necessary to perform boost radiotherapy in patients with bulky unresectable primary tumors or M1LYM because the radiotherapy beam paths to tumors overlapped with the spinal cord. However, these modifications unfortunately resulted in inadequate target coverage. This could have been the cause of poor LRC rates in patients with an unresectable status or M1LYM.

Differently from 3DCRT, intensity-modulated radiotherapy delivered adequate doses in patient even with bulky unresectable primary tumors and M1LYM which were anatomically difficult condition of CESCC [26]. Two retrospective studies suggested that intensity-modulated radiotherapy for CESCC improved LRC [13] and OS rates [9] compared with 3DCRT. Now multi-institutional phase II study using intensity-modulated radiotherapy for CESCC is ongoing (UMIN000009880). Intensity-modulated radiotherapy is one of the techniques which may improve LRC for CESCC in the future.

The long-term follow-up revealed a high incidence of radiation-induced hypothyroidism after definitive radiotherapy for CESCC. The incidence of radiation-induced hypothyroidism was mentioned in three previous reports on radiotherapy for CESCC [2, 12, 13]. Cao [12] reported that four (2.5%) patients had hypothyroidism requiring a lifelong thyroxine replacement. Yamada et al. [2] reported that five among eight patients who survived over 2 years had hypothyroidism. One study with a short follow-up underestimated the incidence of radiation-induced hypothyroidism claiming that no patients had developed it [13].

The thyroid gland is located in the same axial level as the cervical esophagus. Contrary to thoracic esophageal carcinoma, radiotherapy fields for CESCC encompassed the whole thyroid with high doses. The doses usually exceeded the threshold dose of radiation-induced hypothyroidism [27]. Hypothyroidism influences the lipid profile and cardiac function, which possibly increases the risk of cardiovascular disease, mortality, and adversely affects daily life. Radiation oncologists should be mindful of radiation-induced hypothyroidism as a late toxicity event and routinely exam the thyroid function during follow-up of patients with CESCC undergoing radiotherapy.

The 10-year cumulative incidence rates of ≥grade 3 cardiovascular disease was 21.3% in the current study. They were not typically well-known radiotherapy-related heart diseases such as pericarditis or pericardial effusion, which occurred 6–12 months after radiotherapy in patients with thoracic esophageal carcinoma [18, 28]. Radiotherapy fields for CESCC did not include the heart. Thus, the cardiovascular events in the current study were unlikely to be associated with radiotherapy itself. The cardiovascular diseases after radiotherapy for CESCC were not mentioned except one report [1], although patients with CESCC possibly have a risk of developing cardiovascular disease because of their lifestyle, similar to patients with squamous cell carcinoma of the head and neck [29]. The current long-term follow-up study suggested that the incidence of cardiovascular events was not particularly low in long-term survivors cured by definitive radiotherapy.

The limitations of this study include that it was a single institutional retrospective study with a limited number of patients and a variety of treatment details (chemotherapy regimens and radiotherapy doses and fields). Reported prognostic factors, such as weight loss and hoarseness, could not be included because of a lack of information. An overestimation of the safety and effectiveness of radiotherapy for CESCC needs to be considered. Future studies with a larger population are necessary to confirm the safety and effectiveness of radiotherapy for CESCC for an extended period.

## Conclusions

Definitive radiotherapy using 3DCRT yielded a cure for patients with CESCC while preserving their laryngopharyngeal function. In locally advanced tumors, an adequate dose delivery to PTVs was difficult using 3DCRT. The control of GTVs remained a challenging issue for a cure. Based on previous data, we performed intensity-modulated radiotherapy on patients with an advanced T status or non-regional lymph nodal metastasis to improve their LRC rates. The current long-term follow-up revealed that the incidence of radiation-induced hypothyroidism and cardiovascular disease was not particularly low. Those with late toxicity events should be cared for as long-term survivors after radiotherapy for CESCC.

## Abbreviations

3DCRT: three-dimensional conformal radiotherapy
CESCC: Cervical esophageal squamous cell carcinoma
CI: Confidence interval
CT: Computed tomography
CTVlym: Clinical target volume of the metastatic lymph nodes
CTVp: Clinical target volume of the primary tumor
ENI: Elective nodal irradiation
GTVs: Gross target volumes
HR: Hazard ratio
IQR: Interquartile range
LRC: Locoregional control
M1LYM: Non-regional lymph nodal metastasis
OS: Overall survival
PS: Performance status
PTV: Planning target volume
